# Long-term increases in soil carbon due to ecosystem fertilization by atmospheric nitrogen deposition demonstrated by regional-scale modelling and observations

**DOI:** 10.1038/s41598-017-02002-w

**Published:** 2017-05-15

**Authors:** E. Tipping, J. A. C. Davies, P. A. Henrys, G. J. D. Kirk, A. Lilly, U. Dragosits, E. J. Carnell, A. J. Dore, M. A. Sutton, S. J. Tomlinson

**Affiliations:** 1 Centre for Ecology and Hydrology, Lancaster, LA1 4AP UK; 2 Lancaster Environment Centre, Lancaster University, Lancaster, LA1 4YQ UK; 3 Cranfield University, Bedford, MK43 0AL UK; 4 James Hutton Institute, Aberdeen, AB15 8QH UK; 5 Centre for Ecology and Hydrology, Edinburgh, EH26 0QB UK

## Abstract

Fertilization of nitrogen (N)-limited ecosystems by anthropogenic atmospheric nitrogen deposition (N_dep_) may promote CO_2_ removal from the atmosphere, thereby buffering human effects on global radiative forcing. We used the biogeochemical ecosystem model N14CP, which considers interactions among C (carbon), N and P (phosphorus), driven by a new reconstruction of historical N_dep_, to assess the responses of soil organic carbon (SOC) stocks in British semi-natural landscapes to anthropogenic change. We calculate that increased net primary production due to N_dep_ has enhanced detrital inputs of C to soils, causing an average increase of 1.2 kgCm^−2^ (c. 10%) in soil SOC over the period 1750–2010. The simulation results are consistent with observed changes in topsoil SOC concentration in the late 20^th^ Century, derived from sample-resample measurements at nearly 2000 field sites. More than half (57%) of the additional topsoil SOC is predicted to have a short turnover time (c. 20 years), and will therefore be sensitive to future changes in N_dep_. The results are the first to validate model predictions of N_dep_ effects against observations of SOC at a regional field scale. They demonstrate the importance of long-term macronutrient interactions and the transitory nature of soil responses in the terrestrial C cycle.

## Introduction

Soil organic matter (SOM) is a key ecosystem component, both as a store of carbon that can exchange with the atmosphere thereby affecting climate^1^, and because of its key soil functional roles in water and heat retention, nutrient cycling, and sorption of contaminants^2^. Its source is predominantly plant material (litterfall, decaying roots, root exudates), the supply of which might increase where net primary production (NPP) is enhanced by the fertilizing effect of atmospheric N deposition^3–5^. The latter originates from a wide range of emission sources, and comprises oxidised N (NO_x_) and reduced N (NH_x_). Whereas NO_x_ is mainly due to combustion processes such as those occurring in power generation, industry and motorised transport (including shipping), most ammonia (NH_3_) emissions originate from agricultural practices including the management of livestock manures and slurries, and mineral fertiliser application^6^.

**Figure 1:**
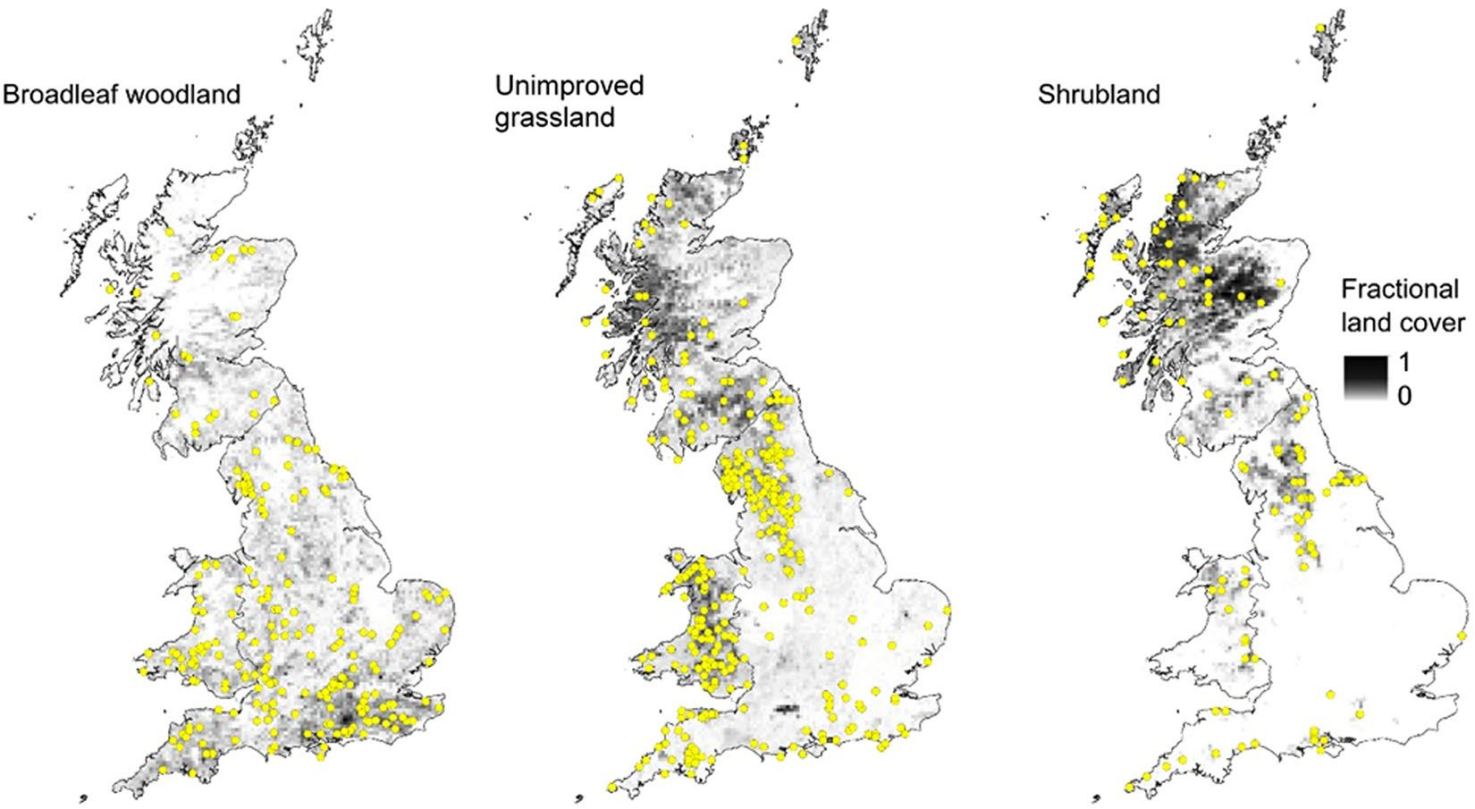
Maps showing the occurrence of the three semi-natural habitat types (grey scale) and sampling locations (circles). The fractional land cover refers to individual 5 km × 5 km grid cells used in simulation modelling. The maps were derived and plotted by aggregating land cover mapped at 25 m × 25 m resolution in the UK Land Cover Map 2007^33^, using ArcGIS 10.4 (http://desktop.arcgis.com/en/arcmap/) (accessed 09/11/2016).

Whereas there is convincing evidence from both experimental manipulations and field observations that forest growth responds positively to N_dep_^7–9^, evidence for a consequent SOC response comes mainly from short-term experimental studies, almost all at high N inputs compared with ambient values^9^, and from modelled estimates^10, 11^. One field study is that of Kirby *et al*.^12^ who reported that for British woodlands, increases in the SOM of mineral and organo-mineral soils between 1971 and 2001 were positively correlated with changes in modelled N_dep_. Data for other ecosystem types are sparse^10^. To explore possible long-term and widespread SOC response to N_dep_ in both forest and non-forest ecosystems, we conducted a combined modelling and data analysis of the soils of British semi-natural ecosystems comprising broadleaf woodland, unimproved grasslands, and shrublands, which account for a total area of 6.8 × 10^4^ km^2^, i.e. 32.5% of Great Britain (Fig. 1). These ecosystems are well-suited to a large-scale evaluation in view of the wide range of N_dep_ (1^st^ to 99^th^ percentile 0.2–3.6 gNm^−2^ a^−1^ in 2010) and the wealth of data available from repeated soil sampling. The modelling was done by combining the ecosystem model N14CP^13^ with a new high-resolution spatio-temporal N_dep_ dataset covering the period since 1750, produced through atmospheric emission, transport and deposition modelling.

## Results

We calculated how SOC pools evolved in Great Britain over the Holocene using the ecosystem model N14CP which links the plant-soil C, N and phosphorus (P) cycles, and has been parameterised and tested with plot-scale data for sites across N Europe^13^ (see Methods). The model was driven by national climate, vegetation and soil datasets, modelled atmospheric deposition (see Methods) and estimated vegetation history. It was implemented for 5 km × 5 km grid cells. A key modelling assumption is that NPP depends on a single limiting factor, temperature, rainfall, N supply or P supply; for these Btirish sites, all locations were found to have been continuously N limited. In blind comparisons with data, the simulations produced reasonable estimates of SOC pools (see Methods).

**Figure 2:**
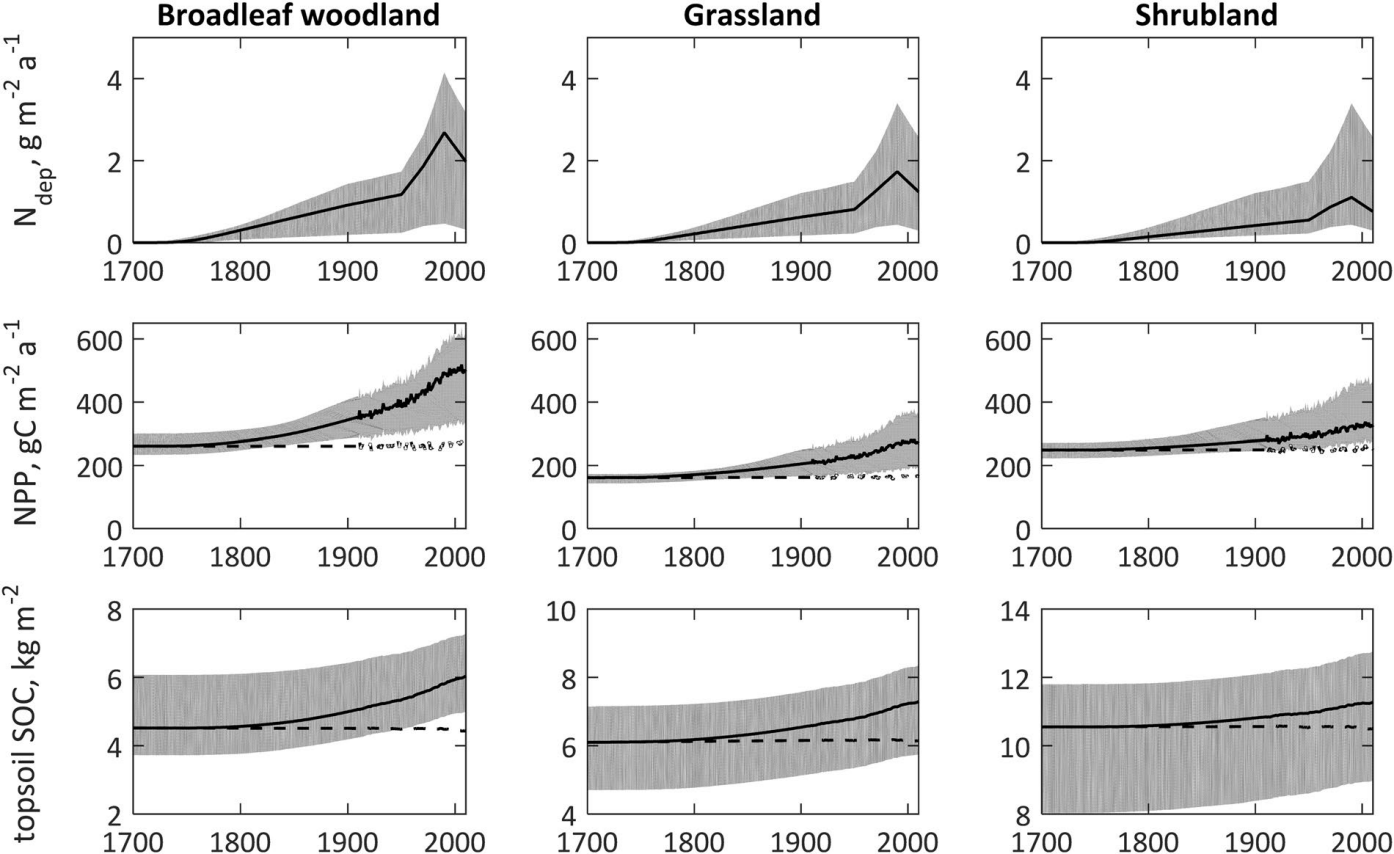
Simulated changes in atmospheric nitrogen deposition (N_dep_), net primary productivity (NPP) and soil organic carbon (SOC) over the period 1700–2010 for semi-natural land areas of Great Britain. The central line is the mean, ranges are 5 and 95%-iles. Dashed lines show mean results with N_dep_ set to zero.

**Figure 3:**
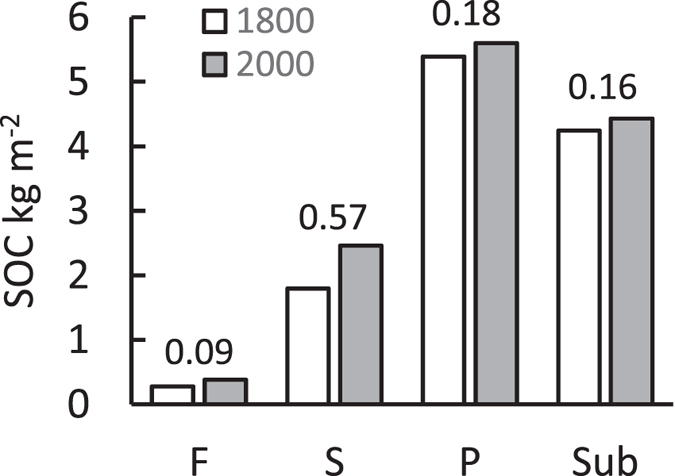
Simulated soil organic carbon (SOC) pools in 1800 and 2000; average values weighted by area of broadleaf woodland, grassland, and shrubland in Great Britain. Key: fast (F, mean residence time 1 yr), slow (S, c. 20 yr), passive (P, c. 1000 yr) and subsoil (Sub, c. 2000 yr). The values above the pairs of bars show the fractional contributions to the total increase in SOC.

The changes in anthropogenic N_dep_ (Fig. 2, top row) caused increases in NPP (Fig. 2, middle row), by an average of 110 gCm^−2 ^a^−1^ (50%), and therefore a greater supply of organic C to the soil and enhanced SOC pools (Fig. 2, bottom row). The additional amounts of SOC from 1750 to 2010 were 21, 38 and 20 Mt for broadleaf woodland, unimproved grassland and shrubland respectively, equivalent to an average increment of 1.2 kgCm^−2^ or an average annual accumulation of 4.6 gCm^−2 ^a^−1^. From an analysis of model performance (see Methods), it can be shown that uncertainty in these SOC increases arises primarily from uncertainty in N_dep_, and it is reasonable to assume an error of c. 30% in the estimated values. The extra SOC was spread through four model pools, fast (mean residence time 1 yr), slow (c. 20 yr), passive (c. 1000 yr) and subsoil (c. 2000 yr), with more than half (57%) accumulating in the slow pool (Fig. 3). The average calculated ratio of SOC gained to N_dep_ for all the soils considered over the entire period was 8.2 gCgN^−1^, at the lower end of the range suggested by de Vries *et al*.^10^. Enrichment of the ecosystems by N_dep_ was calculated to affect total denitrification (i.e. gaseous loss of both nitrous oxide, N_2_O, and dinitrogen, N_2_), such that over the period 1750–2010, instead of 32 gNm^−2^ being lost to the atmosphere, the total was 40 gNm^−2^, a net increase of 8 gNm^−2^.

The model predictions of increased SOC were tested using data for 1911 field sites (Fig. 1) where topsoils had been sampled and then resampled, on average 27 years later, during the period 1959 to 2010 (Tables 1 and 2; see Methods). The measured SOC variable was concentration, expressed as % by weight. At each site, the model-simulated change in SOC pool (gCm^−2^) was converted to a simulated change in SOC concentration (%C) by assuming that the average simulated SOC concentration was the same as the average of the measured values. This took into account both concentration change and soil thickening that could arise from increased organic matter input (see Methods). To evaluate the model’s prediction of SOC change, we compared values of *R*_SF_, the ratio of topsoil SOC concentration at the second sampling to that at the first (see Methods), transforming the data logarithmically to reduce the skew in the data and hence the influence of extreme values. We combined data for soils under grass and shrubs into a single data set, and analysed them separately from those for the woodland soils.

**Table 1:** Summary of topsoil resampling sites.

Data source	Locations^a^	Year of 1^st^ sample	Year of 2^nd^ sample	Mean time period, yr	Number of sites			
					Broadleaf woodland	Unimproved grassland	Shrubland	Total
National Soil Inventory	E, W	1978–1983	2003	22	95	199	54	348
Countryside Survey 1	E, S, W	1978	1998	20	11	137	56	204
Countryside Survey 2	E, S, W	1978	2007	29	13	146	63	222
British Woodland Survey	E, S, W	1971	2001	30	1106	—	—	1106
National Soil Inventory of Scotland & Rare Soils	S	1959–1988	2007–2010	27	—	13	18	31
Total					1225	495	191	1911

^a^E- England, S- Scotland, W- Wales.

**Table 2:** Topsoil SOC data, 5–95 percentiles of %C.

	Sampling	Broadleaf woodland	Unimproved grassland	Shrubland
National Soil Inventory	1	2.2–17.4	2.9–47.2	4.5–54.1
	2	2.1–10.5	2.9–42.2	4.4–47.0
Countryside Survey 1	1	2.2–11.8	2.8–43.5	9.5–51.6
	2	3.5–19.7	3.6–48.9	15.1–53.0
Countryside Survey 2	1	2.2–10.1	2.8–43.5	5.2–51.7
	2	3.1–20.2	2.9–38.3	10.5–52.3
British Woodland Survey	1	3.4–24.4	—	—
	2	3.5–26.5	—	—
National Soil Inventory of Scotland & Rare Soils	1	—	4.5–48.9	12.6–49.9
	2	—	6.2–47.5	13.7–48.1

Over the full range of SOC concentration (0.6–53%), simulated and observed average *R*_SF_ values for woodland soils are similar, and the observed value is significantly (P < 0.01) greater than 1.0 (Table 3a), indicating significant increases in SOC concentrations over the observation period. The confidence intervals for the observations are inevitably wider than those for the modelled values, owing to the large scatter in the observed data, most of which results from local spatial soil heterogeneity, which is not captured by the model. For all soils under unimproved grassland and shrubland, an average value of *R*_SF_ greater than 1.0 was predicted by the N14CP model, whereas the observations give a value slightly, although not significantly, less than 1.0. A clearer result is obtained by restricting the analysis to soils with relatively low SOC concentrations, since in such soils changes in the C pool are mainly seen as changes in SOC concentration, rather than as soil thickening. When this is done (Table 3b), simulated and observed average *R*_SF_ values remain similar for woodland soils, but now there is better agreement for soils under unimproved grassland and shrubland, and the observed means for both vegetation groups are significantly greater than 1.0.

**Table 3:** Calculated and observed values of *R*_SF_, the ratio of the soil organic carbon (SOC) concentration at the second sampling to that at the first, with 95% confidence intervals intervals, for topsoils of Great Britain, based on sampling between 1959 and 2010 (Table 1).

	Calculated	Observed
(*a*) *all soils*		
broadleaf	1.052 ± 0.003	1.053 ± 0.035**
grass & shrub	1.022 ± 0.002	0.989 ± 0.044^*NS*^
(*b*) *soils with [SOC]* < *10%*		
broadleaf	1.054 ± 0.004	1.052 ± 0.037***
grass & shrub	1.032 ± 0.004	1.052 ± 0.046*

For the observed ratios, significant difference from 1.0 is indicated: *NS* not significant, *P ≤ 0.05, **P ≤ 0.01, ***P ≤ 0.001.

A second test of the model is whether the data show significant spatial differences in relation to N_dep_. Again, this is best considered only for the soils with SOC <10%, to focus on conditions where variations in SOC pool are reflected by variations in SOC concentration. Regression slopes of simulated and observed log_10_ *R*_SF_ against N_dep_ (Table 4) are in agreement, although the observed slope for soils under improved grassland and shrubland is not significant.

**Table 4:** Calculated and observed slopes of spatial regressions of log_10_ *R*_SF_ (ratio of SOC concentration in second to first soil sampling) against N_dep_ (gNm^−2^ a^−1^) in 2000 at 5 km grid resolution across Great Britain.

	*n*	Calculated	Observed
broadleaf	864	+0.0091 ± 0.0032	+0.0112 ± 0.0060***
grass & shrub	338	+0.0055 ± 0.0023	+0.0056 ± 0.0104^*NS*^

The number of sites is indicated by *n*, error values are 95% confidence intervals. Whether the observed slope is significantly different from zero is shown by *NS* (not significant) and ***(P ≤ 0.001).

## Discussion

The present study used a large dataset for British semi-natural soils, which we generated by combining several smaller datsets (Table 1). Previous evaluations of SOC change using the individual datasets gave less clear-cut results, including decrease or increase in SOC depending upon the range of SOC concentration considered^14, 15^ and little or no change^12, 16, 17^. Our results are more definite because of (a) the larger sample size, (b) the omission of coniferous woodland which is mostly newly-planted and managed, (c) the use of *R*_SF_ as the diagnostic variable, and (d) our focus on soils with relatively low SOC concentrations.

Overall, the observations provide strong quantitative support for the model prediction of a general increase in the SOC contents of these soils, and the role of N_dep_. The signal is stronger for woodland soils than those under grass and shrubs, because the scavenging effects of trees enhance N_dep_ inputs^18^ so that trees receive proportionally greater extra N. Moreover, the fewer data for soils under unimproved grassland and shrubland make it harder to achieve statistical significance.

The underlying cause of the net accumulation of SOC simulated by the N14CP model is the enrichment of the ecosystem by atmospherically-deposited N, which enhances the flux of plant available N, so that, because N is the limiting factor for plant growth, there is an increase in NPP. In turn, litter production and the supply of carbon to the soil rise, enlarging all three topsoil SOC pools and also subsoil SOC. In the ecosystem transition brought about by N deposition, the SOC pools grow in inverse proportion to their turnover rates, but because the slow pool is relatively large (Fig. 3) it shows the largest absolute SOC increase (Fig. 3). The soil organic N pools grow, more-or-less in parallel with the SOC, so that the simulated soil C:N ratio remains about the same throughout the enrichment^13^. The model omits other possible large-scale determinants of SOC change such as the effects of increased N on litter decomposition rates^9, 19, 20^, fertilization by increasing CO_2_^21^, changes in forest management^12, 22^, and reductions in NPP due to ozone toxicity^23^, which may mean that at least in the long term these effects are quantitatively less important than fertilization by N_dep_, and/or that their effects largely cancel each other. Although the parameterisation includes the effects of pH on SOM turnover, the parameter values are such that there is little pH dependence, and consequently little effect on SOC of recent widespread increasing pH in British soils^12, 16, 24^ is simulated.

Possible climatic effects are included in N14CP via the effect of temperature on maximum NPP, but more importantly by the assumed positive temperature dependences of metabolic reactions, notably soil organic matter turnover rates. Temperature effects alone can be gauged by the simulations for zero N_dep_ in Fig. 2 (dashed lines in the middle and bottom panels). Minor increases in NPP are predicted owing to greater N availability resulting from faster organic matter turnover, but the resulting increases in detrital additions to soil organic matter are more than offset by increased turnover rates, leading to slight declines in SOC. However these effects are small in comparison with the large decreases in SOC (mean ~70 gCm^−2^ a^−1^) reported over the period 1987/88 to 2011 for 24 forest sites in the German Alps^25^, and attributed to faster SOC turnover due to increased summer temperature (mean 0.49 K decade^−1^) and changes in snow cover and freeze-thaw cycles associated with climate change. A loss rate of such magnitude due to temperature change cannot be simulated with N14CP; our calculated rate of SOC accumulation (mainly due to N_dep_) of 13 gm^−2 ^a^−1^ for broadleaf woodland over the period 1971–2007 is more compatible with the results of a study^26^ covering the whole of Germany (1800 resampled sites) for the period 1987/1992 to 2006/2008, in which an increased forest SOC of 41 gm^−2 ^a^−1^ was found.

As well as its effects on SOC, fertilization by N_dep_ can also promote the storage of organic carbon in woody biomass^9–11^. However for Great Britain such storage is limited firstly by the relatively small area of broadleaf woodland, c. 20% of the total area of the semi-natural ecosystems considered here, and secondly because of the removal and burning of wood. Carbon storage in grass and shrub biomass is inconsequential. Therefore the effects of N_dep_ on carbon storage in Great Britain are mainly through SOC. The estimated increased SOC storage calculated here totals 78 Mt, which is equivalent to about 0.5% of the total anthropogenic emissions of CO_2_-C from Great Britain over the same period (1750–2010). The high population density of Great Britain means that its anthropogenic CO_2_-C emissions per unit area are relatively large, and its small area of woodland limits biomass storage, and therefore at bigger scales, European or global, the proportional effect of N_dep_ on C storage is likely to have been appreciably greater.

The accumulated SOC cannot be regarded as permanently sequestered. To maintain present levels, or to increase them, continual inputs of litter at the same rate are needed, which means that the elevated NPP must be maintained. Future declines in N_dep_ would eventually lead to decreased NPP, the litter input would be reduced, and CO_2_-C losses by decomposition would become greater than litter C inputs, with a net return of CO_2_-C to the atmosphere. As noted above, much (57%) of the C calculated to have been gained by the soils studied here over the period 1750 to 2010 is mainly in the 20-year pool (Fig. 3), which means that the soils respond fairly quickly to changes in N_dep_. This is shown by the fairly rapid accumulation of SOC since 1750, and by the recent lower rate of of increase in modelled SOC, reflecting reductions in N_dep_ since 1990 (Fig. 2). In terms of greenhouse gas balance, the release of N_2_O from the soils as part of the increased denitrification that accompanies N enrichment due to N_dep_ will have partially offset the CO_2_-C sequestration, but this too depends upon changes in N_dep_. These dynamic relationships, and the transitory nature of soil responses, need to be factored into predictions of long term changes in the regional and global cycles of C and N.

## Methods

### Modelling long-term spatially-distributed N_dep_

Atmospheric nitrogen deposition (N_dep_) was modelled for a series of time steps between 1800 and the present (2010), at a 5 km × 5 km grid resolution. The N deposition maps were derived by modelling the magnitude and spatial distribution of emissions of oxides of nitrogen (NO_x_) and ammonia (NH_3_) for the historical time period, and running an atmospheric chemistry and transport model (FRAME; Fine Resolution Atmospheric Multi-pollutant Exchange) to estimate total N deposition for the years 1800, 1900, 1950, 1970, 1990 and 2010. Sulphur dioxide (SO_2_) emissions, chemistry and deposition were also modelled for the same years, as they impact on the chemical transformations in the atmosphere and therefore the lifetime and transport distance of nitrogen-containing gases and particles before deposition.

Emissions of NO_x_, NH_3_ and SO_2_ originate from a wide range of sources. Oxidised N and SO_2_ emissions are mainly due to combustion processes such as those occurring in the power generation and industry sectors, as well as from domestic combustion and motorised transport (including shipping etc.). Ammonia, on the other hand, mainly originates from agricultural sources such as livestock manures and slurries as well as mineral fertiliser application (83% of total 2014 UK NH_3_ emissions^6^), with waste processing, domestic pets and horses and natural sources etc. also contributing. Combustion sources are also estimated to contribute to NH_3_ emissions significantly on a global scale^27, 28^, though for historical emissions for the UK the main uncertainty concerns fuel source emissions from domestic coal burning^29, 30^. Spatial datasets are normally produced by combining activity data (i.e. emission source populations or process locations and activities) with emission factors (i.e. average source strength of emissions per unit of activity data).

The emission maps were derived through spatial modelling, using a combination of spatial and non-spatial data, and a number of different approaches for different emission source sectors. For agricultural NH_3_ emissions, the AENEID model^31, 32^ was used, which combines agricultural livestock and crop statistics with land cover and agricultural practice data to derive high spatial resolution emission maps^33–38^. The model was adapted for different historic periods, to account for changes over time in the zonal aggregation and livestock and crop categories recorded in the available agricultural statistics parish, county, rural district level datasets, which also differed between the four countries of the UK, i.e. England, Wales, Scotland, Northern Ireland^39, 40^. In terms of emission factors, the latest UK estimates for 2010 and back-cast to 1990 from the national inventory (NAEI) were used, with data for earlier years adapted to historic conditions, taking into account fertiliser N input to crops and grassland^41, 42^, N content in livestock excreta^43–47^, milk yield of dairy cows (highly correlated with N excretion rates^47^), changing livestock weights (e.g. increases in size due to animal breeding and feeding advances etc. ref. 48). For activity statistics and emission factors for combustion sources, available modern national inventory estimates and back-cast timelines from the NAEI were used for the more recent period (2010, 1990, 1970), with historic time series of power station fuel use, road transport statistics, human population census data, etc. used to extend the time series back to 1800^49–58^.

Substantial NH_3_ emissions from domestic coal combustion had previously been estimated for the UK^29^ at 40, 110, 80 and 5 kt of NH_3_-N for 1850, 1900, 1950 and 1990, respectively, while recognizing a shortage of NH_3_ emission measurements from this source. For 1900, this source amounted to around 70% of total estimated UK NH_3_ emissions. To address this uncertainty, a specific campaign of measurements of N emissions from domestic coal burning was conducted (to be reported elsewhere), which was not able to support these large estimates. Measured emission rates of NO_x_ in this campaign were consistent with the inventory estimates, and the latter were used, because they are based on a larger dataset. For NH_3_, a much lower emission rate of 4.5 g NH_3_-N per tonne of coal burnt was used, based on the new measurements, which is consistent with a total UK NH_3_-N emission from coal of 0.15 kt NH_3_-N for 1900. While much smaller estimates of NH_3_ emission from this source were supported by these measurements as compared with ref. 29, it is recognized that burning sources of NH_3_ emission remain a major source of uncertainty^30, 59^.

Long-term land-cover changes were incorporated based on the current UK Land Cover Map 2007^33^ and the mapping by Dudley Stamp and colleagues in the 1930s^60^, with changes such as large areas of afforestation (e.g. Kielder Forest, northern England) influencing emission distributions and N deposition regionally over the historic period. For all datasets, careful checking and gap filling was necessary, taking account of historic changes in boundary maps (e.g. county boundaries), and a key consideration was the development of a consistent multi-year gridded dataset that was suitable for analysis, interpretation and comparisons across the time period.

The emission maps, together with land cover and meteorological data, were used as input to the UK FRAME atmospheric chemistry transport model to generate estimates of nitrogen and sulphur deposition. FRAME is a Lagrangian model run at a 1 km^61^ or 5 km^62^ grid resolution over the British Isles which simulates the emissions, transport, chemical transformation of gases and particles as well as dry and wet deposition of nitrogen and sulphur compounds. The boundary conditions for air concentrations used in the regional simulation were generated from a European scale simulation run with a 50 km grid resolution using a time series of European emission maps for 1900–2010 (ref. 63 and Simpson D., Chalmers University of Technology, Gothenburg, Sweden, pers. comm.). The performance of the FRAME model has been evaluated by comparison with measurements of gas and aerosol concentrations as well as concentrations in precipitation from national monitoring networks^61, 64^. The model was able to satisfy the criteria of fitness for purposes required for use in policy applications and has been used for future emissions scenario modelling and the assessment of impacts on natural ecosystems^65, 66^. For this study, long-term average meteorological conditions were used rather than specific annual data, to keep the long-term trends comparable, rather than having to take specific annual conditions into account when interpreting a 200+ year time series, with the model run at a 5 km grid resolution. The deposition datasets were compared against measurement based data^67^ (wet deposition, gaseous and aerosol concentrations) for the recent period where national monitoring networks exist (1990 and 2010), with a good fit. However, for the historic period (1800 to 1950), suitable measurements are very scarce^29^.

The gridded population estimates (1801–1951) created for use here are based on data provided through www.VisionofBritain.org.uk, using historical material which has been re-districted by the Linking Censuses through Time system, created as part of ESRC AwardH507255151, by Danny Dorling, David Martin and Richard Mitchell. The gridded population estimates (1971–2011) are based on data provided with the support of the ESRC and JISC, and use boundary material which is copyright of the Crown and the EDLINE Consortium data provided with the support of the ESRC and JISC. The agricultural livestock and crop maps for 1970–2010 were created with data from the EDINA AgCensus database http://edina.ac.uk/agcensus/ at Edinburgh University Data Library and DEFRA for England, The Welsh Assembly Government for Wales, and The Scottish Government (formerly SEERAD) for Scotland.

### Ecosystem modelling with N14CP

The N14CP ecosystem model^13^ simulates the inter-linked cycles of C, N and P in plant-soil systems, starting at the onset of the Holocene, on a quarter-year time step. Four types of vegetation are considered, namely broadleaved woodland, coniferous woodland, herbs (principally rough, neutral, calcareous and acid grasslands) and shrubs (heather, heather grassland and montane habitats), each of which has two end-member stoichiometric compositions.

NPP is calculated on the basis of a limiting factor, potentially temperature, rainfall, available N or available P. For each location and year, total rainfall and mean temperature values are first used to estimate NPP without nutrient limitation. Plant N and P demands to achieve this NPP are then compared with calculated available N and P from SOM turnover and stored plant pools. If the available nutrients cannot meet the NPP demand, then whichever has the lower relative availability is considered to be the limiting nutrient.

SOC is generated by the entry into the soil of plant detritus comprising above-ground leaves and twigs, dead roots and root exudates. Larger woody components (boles and branches) are assumed to decompose on the soil surface, releasing CO_2_ to the atmosphere but N and P to the soil. The SOM is partitioned among pools with turnover rates of c. 1 year (fast), c. 20 years (slow) and c. 1000 years (passive); the exact turnover rates depend upon temperature. The model is driven by climate, atmospheric deposition and soil type, and simple estimates are made about vegetation history. Key assumptions about the behaviour of N are (a) that N fixation depends upon the availability of P, and (b) that there is down-regulation of N fixation by anthropogenic N_dep_, which means that during the last 200–300 years N_dep_ has become the dominant source of nutrient N to natural and semi-natural ecosystems in many parts of northern Europe, including Great Britain. The model was calibrated^13^ with data for semi-natural ecosystem sites of northern Europe, and with generic ^14^C data to constrain the contributions of the fast, slow and passive pools.

N14CP was applied to British semi-natural sites on a 5 km × 5 km grid. For the period 1910 to 2011, the model was driven by seasonal temperatures and precipitation^68^. Prior to this, long-term gridded climate averages (1910–1940) were modified by historical anomalies^69^. For the period from 1750, spatially-resolved N_dep_ and S_dep_ were obtained from the deposition model described above with values interpolated linearly between 1750 and the deposition model years of 1800, 1900, 1950, 1970 and 2010. Base cation deposition over time was estimated from values for 2012 from the CBED model^67^, combined with an historical anomaly^13^ to provide time-series inputs. The UK Land Cover Map 2007 (LCM)^33^ was processed to provide fractional covers on this grid scale for the three broad habitat types examined here, i.e. broadleaf woodland, grasslands and shrubs. It was assumed that areas classified today as broadleaf woodland have been so since forests succeeded post-glaciation grasslands, broadly estimated to have been at 6000 BC. Contemporary grasslands were assumed to have been grasslands since forest clearance, dates for which are estimated from data across GB^70^. Shrublands were assumed always to have been shrublands. An initial weatherable P pool (gPm^−2^) was set for each grid square on the basis of two soil types ((i) podzols and rankers, (ii) other soils)^13^, the fractional coverage of each being calculated from gridded soil survey data from the National Soil Resources Institute^71^ and the James Hutton Institute (JHI)^72^. Model outputs, on an annual basis, were the pools of SOC and nutrients (biomass C, N and P, topsoil and subsoil SOC, SON and SOP, P sorbed to soil surfaces), and their fluxes (NPP, N fixation, CO_2_, N lost via denitrification, DOC, DON, DOP, and dissolved inorganic N and P).

### Model performance

To constrain the model parameters and test the model’s performance, we previously^13^ conducted a detailed calibration using plot-scale data for 44 semi-natural ecosystem sites (woodland, grassland, shrubland) of northern Europe, and blind-tested this non-site specific parameter set against a further 44 sites. A comprehensive sensitivity analysis considering parameter interactions identified the parameters which had most influence on the multiple outputs of interest, including NPP, soil C, N and P, and dissolved nutrients. These parameters were calibrated in a combination of multiple local and global searches using NPP, soil C, N and P pools and fluxes, and generic radiocarbon data, and then the model was tested using the same output variables. The results showed that the model successfully simulates and predicts average ecosystem behaviour, but in the absence of site-specific information, e.g. P weathering rate, it cannot distinguish the behaviours of individual sites.

In addition to the site-scale testing^13^, evidence that N14CP can provide reliable predictions is as follows. (1) The model found that N limitation is widespread which agrees with literature data^73^. (2) The model predicts an increase of c. 30 g C in above-ground unmanaged woodland biomass per g N_dep_^−1^, in agreement with the range of 15–40 g C g N^−1^ estimated for European forests^10^. (3) A general assessment of model performance for Great Britain was performed by comparing modelled topsoil C pools with values estimated for a depth of 15 cm from measured SOC concentrations reported by the Countryside Survey for Great Britain (CS)^16, 74^ (see below), by estimating soil bulk density (BD) from %C using an equation derived from CS data (BD = 1.29e^−0.206%C^ + 2.51e^−0.003%C^ − 2.057)^16^. The average simulated topsoil C pool over all the sites on both sampling dates was 6430 gCm^−2^ (standard deviation, sd, 1500 gCm^−2^), in reasonable agreement with the average pool to a depth of 15 cm estimated from the measurements, 7580 gCm^−2^ (sd 1320 gCm^−2^). For soils under different vegetation types, the simulated average soil pools (gCm^−2^) were 5701 (broadleaf woodland), 6842 (grassland) and 9897 (shrubland), in the same sequence as the observed values of 7440, 7505 and 8620 respectively. The average modelled soil C:N ratio for the 398 CS sites with measurements of both C and N was 15.6 (sd 1.2) sampled in 1998 and 2007, in agreement with the measured ratio of 16.2 (sd 7.9). (4) The model is tested further in the present work, by comparison of simulated and observed changes in SOC concentration in the late 20^th^ Century (see Results, Tables 3 and 4).

We explored how uncertainty in key input drivers affects model output by conducting a simple sensitivity analysis. In this analysis, we ran N14CP for 9 randomly chosen example locations covering the 3 vegetation types at the 20^th^, 50^th^ and 80^th^ percentile of cumulative N_dep_ loading between 1750 and 2010. We considered the sensitivity of the soil carbon outputs at these locations to variation in vegetation history, soil type and N_dep_ inputs. The contemporary vegetation at a given location is relatively reliable, but vegetation history is less certain. Therefore we ran the model for different histories, including changes to forest clearance dates and succession (see SI for further details). As described in the preceding section, the fraction coverage of soil types in the grid cells determines the initial P weathering condition. To determine sensitivity to this condition, we varied the soil type between the minimum and maximum P_weath0_ values. Finally, to investigate sensitivity to N_dep_, we varied the input magnitude for each example by ±30%, which encompasses the national scale variation suggested by an inter-comparison of different atmospheric chemistry transport models^64^. In the sensitivity simulations, we considered the one-at-a-time effects of changes to these inputs and the combined effects, requiring 243 model runs.

The results of the sensitivity analysis are shown in Figures S1 and S2. We find that the three input drivers examined have an influence on the magnitude of contemporary carbon stores to varying extents, with shrub locations broadly displaying the greatest sensitivity, which is clearly seen in S1. However, vegetation history and soil type have less influence on the dynamic trends i.e. they have little effect on the change in soil organic carbon over the last ~200 years, which is the focus here. We can conclude that uncertainties in these input drivers do not alter the conclusion that N_dep_ has been a major driver of soil carbon increases for these ecosystem types. The N_dep_ sensitivity results show that the model’s estimates for change in SOC are most sensitive to this input.

### Soils data

Data were compiled for soils under broadleaf woodland, unimproved grasslands and shrubs, since these ecosystems are generally long-established in the UK. None of them is entirely natural, because of relatively light management, but none is significantly fertilized, and they are commonly referred to as “semi-natural”. Conifer sites were excluded because they are predominantly managed woodland undergoing recent (20^th^ Century) land use change. Ombrotrophic peats were excluded because they are nearly exclusively composed of organic matter, and therefore changes in soil C pools cannot be revealed by changes in soil C concentration. Only results referring to the same vegetation on both sampling occasions were used, so as to minimise errors due to site mis-identification. In all cases, soils were sampled from the surface to a measured depth (15 cm in 98% of cases), and any coarse litter on the surface was removed before sampling. The average time difference between samplings was 27 years. Site locations, sampling dates, and numbers of samples are summarized in Table 1, and SOC concentrations in Table 2.

Data from the National Soil Inventory (NSI) came from surveys carried out in England and Wales^14^. The samples were taken to 15 cm depth, bulked from 25 separate soil cores at each site. For both first and second samplings, organic carbon was analysed by the Walkley-Black method at low concentrations (<15% C), and by loss-on-ignition (LOI) for soils with greater %C. Countryside Survey data were obtained from the Centre for Ecology & Hydrology^16, 74, 75^. Single 15 cm deep core samples were taken where possible, shallower samples in cases where a full core could not be taken. Samples from each campaign were analysed for LOI, which was converted to %C using a factor of 0.55^75^. The British Woodland Survey (BWS) soil sampling campaigns^12^ were conducted with the same protocols as CS. Data from the National Soil Inventory of Scotland (NSIS) were taken from sites first sampled in 1978–88 and resampled in 2007–9 by JHI staff. Additionally, we included data from samples taken from soils rare in a Scottish context, initially sampled between 1959 and 1981 and resampled in 2010. The Scottish data referred to excavated pits with sampling by soil horizon, and therefore not to a specified depth. However, sampling depths for the first and second samplings were very similar; on both sampling occasions the range of topsoil depths was from 10 to 24 cm, with an average of 17 cm.

### Estimation of SOC concentrations from simulated SOC pools

We compared simulated and measured changes in SOC in terms of SOC concentration, [SOC] (%C), which was the measured and reported variable in the soil surveys (see above). To relate [SOC] to the simulated SOC pool (*Q*_SOC_, gCm^−2^), we assumed that the topsoil mineral matter pool, *Q*_MM_ (gm^−2^), which is not modelled with N14CP, was constant, and made the approximation that the topsoil was homogeneous. The mean *Q*_SOC_ during the period between two samplings is given by

1 $${Q}_{{\rm{SOC}},{\rm{mean}}}=({Q}_{{\rm{SOC}},1}+{Q}_{{\rm{SOC}},2})/2$$

and the mean simulated SOC concentration (%) by

2 $${[{\rm{SOC}}]}_{{\rm{sim}},{\rm{mean}}}=100\times {Q}_{{\rm{SOC}},{\rm{mean}}}/\{({Q}_{{\rm{SOC}},{\rm{mean}}}/{f}_{{\rm{C}}})+{Q}_{{\rm{MM}}}\}$$

where *f*_C_ is the fraction of SOM that is SOC (taken to be 0.55 in this work). Rearrangement of equation (2) gives

3 $${Q}_{{\rm{MM}}}=\{100\times {Q}_{{\rm{SOC}},{\rm{mean}}}/{[{\rm{SOC}}]}_{{\rm{sim}},{\rm{mean}}}\}-\{{Q}_{{\rm{SOC}},{\rm{mean}}}/{f}_{C}\}$$

which allows the value of *Q*_MM_ to be calculated by assuming that [SOC]_sim,mean_ is equal to the mean of the measured values of [SOC], i.e. [SOC]_obs,1_ and [SOC]_obs,2_, at the two samplings. The simulated values of [SOC] at the first and second samplings are

4 $${[{\rm{SOC}}]}_{{\rm{sim}},1}=100\times {Q}_{{\rm{SOC}},1}/\{({Q}_{{\rm{SOC}},1}/{f}_{{\rm{C}}})+{Q}_{{\rm{MM}}}\}$$

5 $${[{\rm{SOC}}]}_{{\rm{sim}},2}=100\times {Q}_{{\rm{SOC}},2}/\{({Q}_{{\rm{SOC}},2}/{f}_{{\rm{C}}})+{Q}_{{\rm{MM}}}\}$$

We used the variable *R*_SF_ (i.e. [SOC]_2_/[SOC]_1_) to assess change in SOC, to take into account the two ways that a soil can respond to extra C, which are by increasing in C concentration and by thickening. Thus if a soil has low %C, i.e. is high in mineral matter, any additional C mostly goes to increasing the C concentration, whereas a soil with high %C and little mineral matter will respond by thickening, with little or no change in C concentration^17^. If SOC (%) is used as the diagnostic variable, results for the latter type of soil exert a disproportionate influence in the statistical analysis, but the ratio approach avoids this.

### Statistical Methods

Change in SOC was evaluated by modelling the ratio of values at two time points (*R*_SF_) on the logarithmic scale, using a linear mixed effects model^76^. A mixed effects model estimates global fixed effects across the whole system whilst accounting for differences within it by allowing additional sources of error to be included in the model specification. These so called random effects were used here to account for potential differences arising from pooling data across different sites. Similarly data from different habitats could result in random deviances from the global average that are not consistent, i.e. they have the same variance, across habitats. Log_10_ *R*_SF_ was therefore modelled simply using an intercept term to estimate the mean overall change, but incorporating random effects representing different sampling units from which the data are obtained. Analyses were carried out in the R statistical computing environment^77^.

To test whether the mean log_10_ *R*_SF_ differs significantly from zero (i.e. whether mean *R*_SF_ differs from 1.0) one would naturally want to test whether the fitted intercept in the model deviates from zero. However, because the log_10_ *R*_SF_ data cannot be expected to conform to a normal distribution, or any other exponential family distribution, the estimated variance parameters output directly from the model would not be robust enough for hypothesis testing. We therefore adopted a bootstrapping^78^ approach to test the null hypothesis that log_10_ *R*_SF_ = 0. We produced random multiple pseudo-sample sets by re-ordering pairs of SOC values repeatedly (1000 times) and re-estimating the mean log_10_ *R*_SF_. The mixed model described above was then fitted in exactly the same way to each of these sets to produce 1000 estimates of mean log_10_ *R*_SF_, against which the observed change from the original data could be compared. The probability (p) value was then obtained as the percentile amongst the pseudo log_10_ *R*_SF_ values corresponding to the observed log_10_ *R*_SF_.

Testing whether there was a significant relationship between log_10_ *R*_SF_ and N_dep_ was carried out in a similar manner. Linear mixed effects models were used as described above with the addition of N_dep_ as a fixed effect term in the model as well as the intercept term. The random effects structure remained the same and a similar bootstrap procedure was adopted based on the estimated coefficient between log_10_ *R*_SF_ and N_dep_.

## Electronic supplementary material

Sensitivity of N14CP model outputs to input drivers
