# Supplementary material for: Long-term increases in soil carbon due to ecosystem fertilization by atmospheric nitrogen deposition demonstrated by regional-scale modelling and observations
Source: Sci Rep. 2017 May 15;7:1890. doi: 10.1038/s41598-017-02002-w (PMC5432490; doi:10.1038/s41598-017-02002-w)
Supplement: Supplementary file 1 — Sensitivity of N14CP model outputs to input drivers [file 41598_2017_2002_MOESM1_ESM.pdf]

SUPPLEMENTARY INFORMATION

Long-term increases in soil carbon due to ecosystem fertilization by atmospheric nitrogen deposition demonstrated by regional-scale modelling and observations

E Tipping[1*], JAC Davies[2], PA Henrys[1], GJD Kirk[3], A Lilly[4], U Dragosits[5], EJ Carnell[5], AJ Dore[5], MA Sutton[5], SJ Tomlinson[5]

[1]Centre for Ecology and Hydrology, Lancaster LA1 4AP, UK, [2]Lancaster Environment Centre, Lancaster University, LA1 4YQ, UK, [3]Cranfield University, Bedford, MK43 0AL, UK, [4]James Hutton Institute, Aberdeen, AB15 8QH, UK, [5]Centre for Ecology and Hydrology, Edinburgh, EH26 0QB, UK.

Sensitivity of N14CP model outputs to input drivers

Parametric sensitivity was addressed previously[13]. An input sensitivity analysis was undertaken here to assess the sensitivity of the conclusions to the main input drivers. Soil type sensitivity was determined by varying the soil type input variable between its minimum and maximum values[13]. Sensitivity to vegetation history was explored by simulating different scenarios for each plant type. For broadleaf woodland sites, the date of succession from grasslands to trees following glaciation was changed by ± 2000 years spanning 8000 to 4000 BC. The forest clearance date for grassland locations was changed by ± 1000 years. The default history for the shrub vegetation type is uninterrupted shrubland back to the glaciation date, however, there is a possibility that a shrubland has emerged following tree clearance. As such, to test vegetation history, a forested period was inserted in the simulation history between 6000 BC and 3000-1000 BC to represent clearance during the Neolithic/Bronze Age. Sensitivity to atmospheric N deposition was tested by simulating scenarios with ± 30% deposition, applied as a simple multiplier over the whole simulation period. This range encompasses the national scale uncertainty in N deposition[64].

The results of this sensitivity analysis are given in Figures S1 and S2. The time series results in Figure S1 give an indication of the model's sensitivity to the input drivers, considering all combinations of input driver change. To understand the relative sensitivity of each input variable, and in turn the importance of this variable in determining the outputs and supporting the conclusions, the one-at-a-time results are presented in Figure S2. These plots show the sensitivity of the modelled topsoil organic carbon pool in 2010 to the input drivers for the 9 locations across plant type and deposition loadings, and the sensitivity of the change in topsoil organic C between 1750 and 2010, which is the focus period of this study. The results demonstrate that the driver inputs influence the magnitude of the topsoil pools in 2010 to varying extents, with shrub sites being the most sensitive to soil type input and the vegetation history scenarios explored. The latter is unsurprising as the alternative shrub vegetation history scenarios have the most recent changes in vegetation cover. Despite differences in the simulated baseline pool of carbon in the topsoil, vegetation history and soil type have relatively little influence on the *change* in soil organic carbon over the last 200 years, and therefore uncertainty in these variables does not alter the conclusion that fertilization by N deposition has led to long-term changes in SOC in these ecosystems. Variability in N deposition is shown to have the most effect on soil organic carbon pool change.

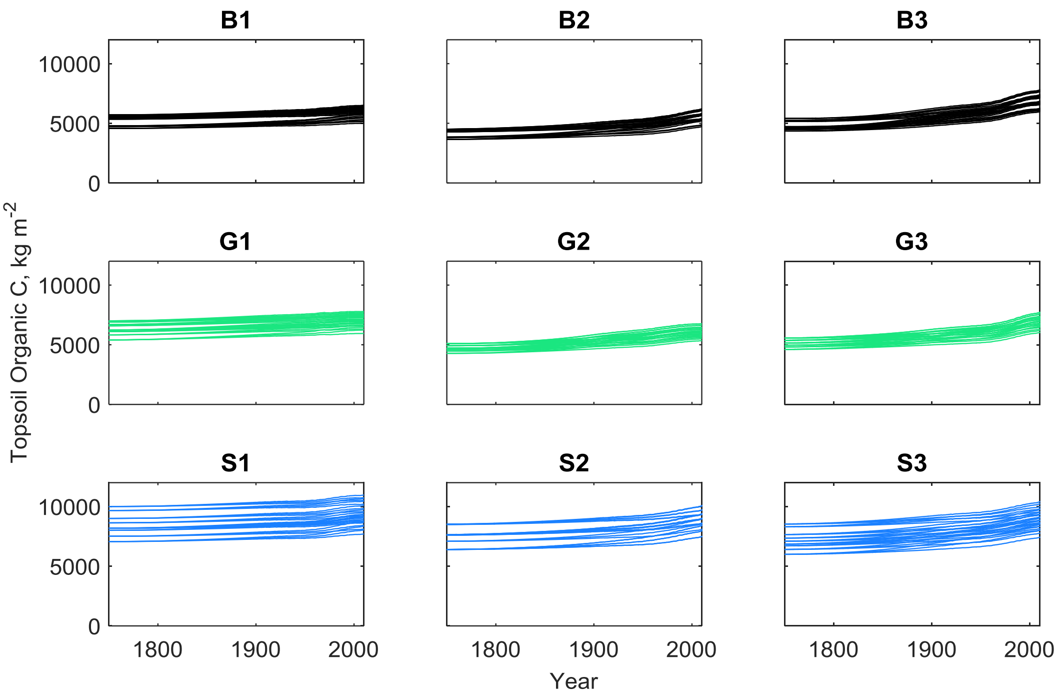

Figure S1 Sensitivity results for nine example locations from the GB wide N14CP simulations. B1-3 are broadleaf locations randomly chosen with cumulative N deposition loadings between 1750 to 2010 at the 20th 50th and 80th percentiles, corresponding to B1, B2 and B3 respectively. G1-3 and S1-3 are the same, but for grass and shrub locations. Model outputs of topsoil organic carbon over time are given for all combinations of the input driver scenarios described comprising 243 model runs (3 locations for 3 vegetation types with 3 Pweath0 values (nominal, minimum and maximum), 3 vegetation history scenarios and 3 N deposition levels (the value at the location ± 30%).

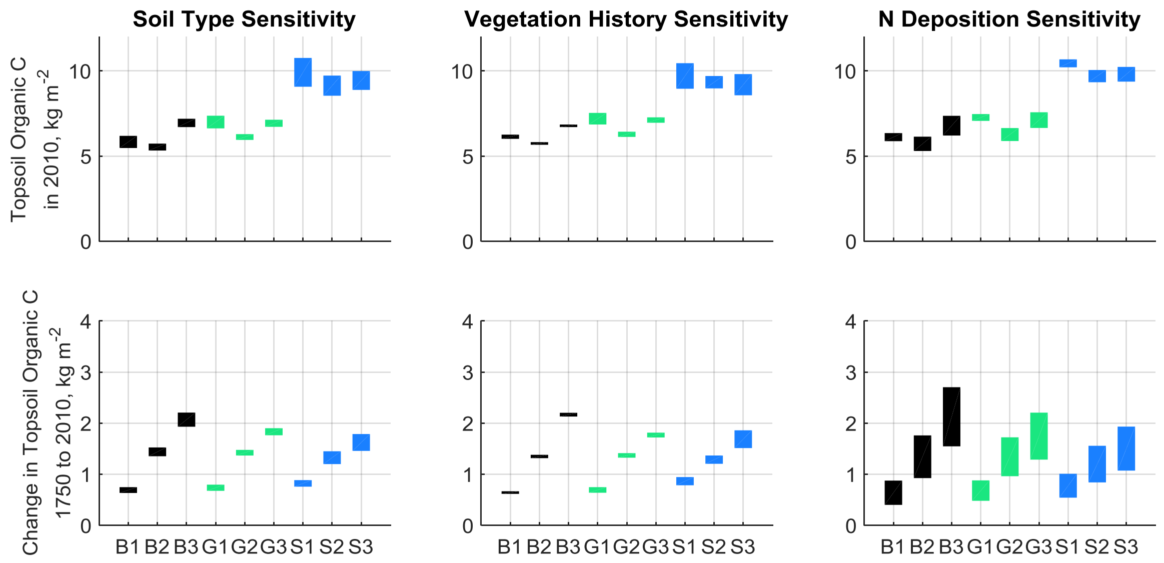

Figure S2 Sensitivity results for nine example locations from the GB wide N14CP simulations. B1-3 are broadleaf locations randomly chosen with cumulative N deposition loadings between 1750 to 2010 at the 20th 50th and 80th percentiles, corresponding to B1, B2 and B3 respectively. G1-3 and S1-3 are the same, but for grass and shrub locations. The top row gives the range of model estimates for topsoil soil organic carbon in 2010 for the nine example locations with varying input drivers. The bottom row gives the range of model estimates for change in topsoil organic carbon pool, as a % of the topsoil pool in 1750.
